# Ethics in pre-ART genetics: a missed X-linked Menkes disease case

**DOI:** 10.1007/s10815-023-02778-z

**Published:** 2023-03-30

**Authors:** A.-M. A. Gerdes, L. Birk Møller, N. Horn

**Affiliations:** Department of Genetics, Copenhagen University Hospital, Rigshospital, Copenhagen, Denmark

**Keywords:** *ATP7A*, Menkes disease, Assisted reproductive technologies (ART), Preconception screening, Variant evaluation, Igenomix

## Abstract

Assisted reproductive technology (ART) has experienced dramatic progress over the last 30 years, and gamete donation is routine in fertility clinics. Major advances in genetic diagnostics are part of this development due to the ability to analyze multiple genes or whole genomes fast and to an affordable prize. This requires knowledge and capability to evaluate genetic variants correctly in a clinical setting. Here we report a Menkes disease case, born after ART, where genetic screening and variant scoring failed to identify an egg donor as carrier of this fatal X-linked disorder. The gene variant is a deletion of a single base pair leading to a frameshift and premature termination of the protein, predicted to result in no or severely diminished function. The variant would be classified as likely pathogenic (class 4) and should be readily detectable by molecular genetic screening techniques. We wish to highlight this case to prevent future similar cases. IVI Igenomix has developed and embarked on an ambitious screening program to detect and prevent a large number of inherited severe childhood disorders in ART pregnancies. The company has recently achieved ISO 15189 certification with competence to evaluate and deliver timely, accurate, and reliable results. Failure to identify a pathogenic variant in the *ATP7A* gene leading to birth of two boys with Menkes disease invokes the required procedures to screen and detect disease-causing gene variants. This calls for ethical and legal considerations in ART diagnostics to prevent fatal errors like the present.

## Introduction

Gene variants are as frequent in gamete donors as in the general population, and inherited conditions occasionally occur in donor-conceived children. Information about circumstances is rarely disclosed except for a few cases [1–3]. Lowe syndrome is an X-linked recessive condition affecting boys, and an unfortunate post-conception discovery of carriership in an egg donor led to counseling and psychological support of the donor, and the remaining cryopreserved embryos being destroyed. Families afflicted were also offered extended medical and psychological care [1]. An exceptional case of transmission of neurofibromatosis type 1 to nine of 23 sperm donor conceived half-siblings led to tightening of legislation [2, 3].

Advances in genetic technologies allow preconception screening to prevent disease in offspring after assisted reproductive technologies (ART) [46]. Fertility clinics use screening programs to test gamete donors to minimize the risk of genetic disorders [4, 5]. Igenomix offers extended screening of genes implicated in severe childhood monogenic disorders, including *ATP7A*. Screening of the partner is included in the pre-ART screening, and in case of a pathogenic gene variant potentially leading to an autosomal recessive disorder, matching is done with a suitable donor, a genetic compatibility test (CGT). Here, we report a Menkes disease case [OMIM #349000] born after ART, where preconception screening and selection failed to identify an egg donor as gene carrier of this fatal X-linked condition.

Menkes disease is a severe childhood disorder caused by pathogenic variants in *ATP7A* [OMIM #300011]. The disease is characterized by progressive neurodegeneration and marked connective tissue anomalies, normally leading to death by 3 years of age. Onset is neonatal or infantile, and symptoms are caused by poor metallation of copper enzymes in secretory pathway [6]. The incidence of diagnosed cases in two large prospective studies was 1:300,000 [7, 8]. An initial retrospective study of five families within 3 years in the Melbourne County gave a higher but uncertain estimate [9]. Recently, a study of *ATP7A* variants in a female cohort in the Genome Aggregation Database (gnomAD) indicated a significantly higher frequency of pathogenic or likely pathogenic variants in the general population, equaling the homologous *ATP7B* gene, causing autosomal recessive Wilson disease [OMIM #277900] [10]. Loss-of-function (LOF) pathogenic variants are common in clinically diagnosed Menkes patients [11] but were found to be rare in the gnomAD female cohort, where potentially disease-causing missense variants are prevalent [10]. *ATP7A* is located on the long arm in a gene-dense region, Xq21.1 near the X-inactivation center, and the mutation rate depends solely on X-chromosomal factors [12]. Therefore, endemic variance does not adhere to the *ATP7A* gene and the incidence is the same worldwide, but diagnosing Menkes disease may vary.

Variants in the *ATP7A* gene can cause milder disease, occipital horn syndrome (OHS) [OMIM #304150], and X-linked motor neuron disease (SMAX3) [OMIM #300489] [13]. Both are much rarer and with later onset of symptoms. OHS is mainly a connective tissue disorder and may be accompanied by intellectual impairment [14, 15]. SMAX3 is an adult-onset progressive motor neuron disease with normal fertility and normal life span [16, 17]. Both OHS and SMAX3 are included in Igenomix screening, but it is not possible to predict disease severity from variants alone [11].

When an *ATP7A* variant is detected, it is important to assess whether it is benign without known disease risk (benign, class 1 and likely benign, class 2), a variant with unknown clinical significance (VUS, class 3), or causes an increased disease risk (likely pathogenic, class 4 or pathogenic, class 5) [18]. Most pathogenic variants in the *ATP7A* gene are novel (70%) [unpubl.], meaning not observed before and therefore unlikely to be reported in any database. Benign variants are more often observed in the background population and recorded in variant databases and in the locus-specific database (LOVD/ATP7A) [11]. It is crucial to discriminate between pathogenic and benign variants. Class 3 variants may require more specific knowledge and eventually lead to reclassification [18–20]. Scoring is not a trivial task, and lack of skills can lead to misclassification of a clearly abnormal finding and failure to diagnose serious illness, like Menkes disease.

## Case story

### Gynecological story

Mrs. AMR (46 years) and Mr. AG (58 years) had failed to conceive for a decade without any known cause before the couple contacted the Valencian Infertility Institute (IVI) in Spain. IVI is a worldwide company with headquarters in Valencia and more than 65 clinics in 12 countries around the world, whereof half in Spain. The ART procedure was performed in a Spanish IVI clinic using an egg donor and husband’s sperm. The pregnancy was complicated by threatened abortion in first trimester (4th week of gestation), and delivery was planned by cesarean section at 39th week of gestation at Hospital San Giovanni connected with the pediatric Hospital Bambino Gesu, Rome. Late May, 2 weeks before planned delivery, the couple was informed about a 50% risk of Menkes disease in their expected boy, and the delivery was accelerated 1 week.

### Clinical history

**Fig. 1:**
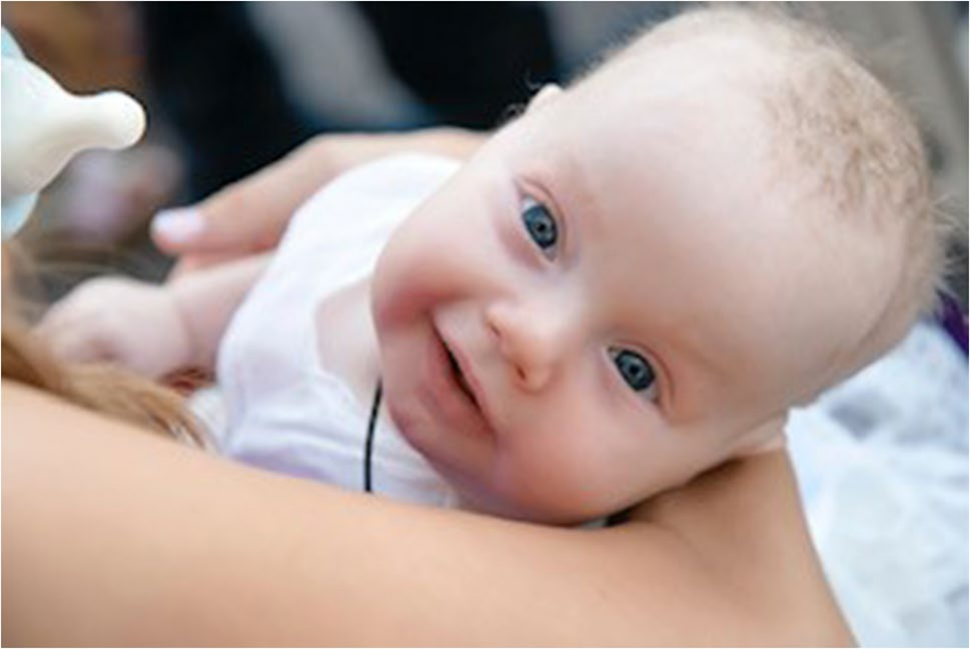
Two-month-old M was an alert little guy with blue eyes, pale skin, pudgy cheeks, and visible hair changes (stubbles)

The boy M was born in June 3, 2020 at 38 weeks gestation. Birth weight was 3.650 kg, and length 51 cm. Day 4, the Menkes diagnosis was genetically confirmed, and subcutaneous copper histidinate treatment was started. At first clinical examination 3 weeks old, M showed minor symptoms. He was alert and reacted adequately, but neurological examination revealed discrete hypotonia and slight lack of head control. Skin and joints were lax, and hair was sparse and depigmented. Seven months old, weight was 5.9 kg and length 65.5 cm, and at clinical examination in July 2021 at nearly 14 months of age, weight was 7.5 kg and length 74 cm. M was still an attentive and curious boy. His hair had grown long but cut short and retained steel wool characteristics and a fairly light reddish color. M suffered from progressive hypotonia but was able to control his head. He was active and responded adequately and followed movements with his eyes. He had no fits. He showed feeding difficulties with prolonged eating time but did not suffer from dysphagia. He had developed respiratory problems (Fig. 1).

## Genetic investigations

### Igenomix

Igenomix is an extension of IVI performing ART research and genetic tests, and includes a growing number of laboratories worldwide that offer preconception screening at different levels. The family opted for the highest level available at the time, Carrier Genetic Test 600 (CGT 600), a test covering more than 600 disorders encoded by 547 genes, most inherited in an autosomal recessive mode, but 27 genes are X-linked recessive, including *ATP7A*. Genes analyzed are listed in an Appendix to the informed consent and on Igenomix website. The genes are screened in a multi-gene panel using whole-exome sequencing (WES) including +/− 10 bp of introns [4, 5]. Variants located in regulatory regions or intronic regions before or after position + 5 and − 5 are not scored [4, 5]. Classification is done using bioinformatics and complies with international guidelines [18]. Identification of X-linked class 4 or 5 variants will lead to exclusion of an egg donor from the egg-donation program.

Seven genes named in the informed consent, on Igenomix website and in publications [4, 5], are tested for a specified number of more common variants, e.g., expansion repeat syndromes and large exon deletions in Duchenne muscular dystrophy. Copy number variants (CNV) are not analyzed in other genes. ART also excludes VUS, and preconception genetic exemptions are de novo mutations including gonadal mosaicism.

CGT 600 testing of the male partner is included in the preconception screening and is performed in precisely the same way [4, 5]. Information on IVI donor-spouse matching is limited, except that it is a blinded computerized process to avoid double heterozygosity of pathogenic autosomal gene variants identified in the male partner. The software tool allows selection of a genetically matched egg donor and depends on availability of a CGT 600 pre-screened egg-donor bank [5].

### Egg donor

The egg donor had given birth to a normal child before entering the donor program, and there was no family history suggesting carriership for a serious, X-linked childhood disorder. Diagnosis of a twin boy with Menkes disease, born in another IVI clinic, led to reassessment of the egg donor.

Twin L, born October 2019, developed disease symptoms, and 7 months old he was diagnosed with Menkes disease. He started copper treatment, but his disease progressed, and he died mid-August 2020, 10 months old.

By May 2020, an *ATP7A* variant had not yet been established in twin L. IVI double checked the egg-donor’s CGT 600 profile, but the genetic report stated that it showed no “genetic variant associated to Menkes disease that was included in the test.” Her screening profile was requested, but not disclosed to the authors of this publication nor the parents. A likely pathogenic *ATP7A* variant in the egg donor was identified by whole gene sequencing, and the genetic counselor stated “as never reported until now, according to scientific databases (ClinVar, HGMD) this variant is not included in the CGT test.” The donor is now blocked to prevent further use and reported to international authorities.

### Affected boy

The class 4 variant NM_000052.7: c.3556delG (p.Glu1186Serfs*3) is a deletion (del) of one base pair in exon 18 leading to a frameshift (fs) and premature termination (*) of the protein. The normal *ATP7A* gene contains 23 exons and encodes 1500 amino acids. The premature stop codon may lead to nonsense-mediated *ATP7A* mRNA decay or alternatively to skipping of exon 18 and a large in-frame deletion (p.Ala1172_Asp1220del). The variant is a LOF variant as the ATP7A protein will lack part or several late domains, and the expected effect is absent protein or a protein with minimal function, but we have not done functional studies.

### Birth mother

IVI’s testing for thrombophilia revealed a heterozygous pathogenic variant in the autosomal gene *MTHFR,* and prophylactic heparin therapy during pregnancy was recommended. The gene variant was not disclosed, nor did Mrs. AMR receive proper genetic counseling related to the finding.

### Biological father

CGT 600 preconception screening showed disease-linked heterozygous gene variants in *CNGB3:* c.1148delC (p.Thr383Ilefs*13) and *ABCA4:* c.2588G>C (p.Gly863Ala). The pathogenic variants were donor matched to avoid double heterozygosity in the fetus and future vision problems.

## Discussion

Although individually rare or ultra-rare, genetic disorders account for a significant proportion of complications in newborns. Igenomix extended preconception screening aims to minimize risk of genetic disease in offspring of gamete donors. X-linked pathogenic variants are established solely by analysis of the egg donor’s screening profile. Despite an alleged high screening level, IVI Igenomix failed to detect a likely pathogenic *ATP7A* variant in the egg donor, and the variant was established only after birth of two affected boys in two different families.

The parents of the present case chose the highest pre-ART screening level, implying a high level for both spouse and egg donor, and Menkes disease is included in Igenomix CGT 600 test [4, 5]. Menkes disease is viewed as ultra-rare [21], although recent data indicate that frequency of potentially pathogenic gene variants in healthy females is more prevalent than anticipated and as common as Wilson disease [10], and preconception screening programs should pay due attention.

Two publications describe how Igenomix screens for variants in 547 genes. For autosomal recessive diseases, egg donors with pathogenic variants are accepted, but to avoid combinations where egg donor and the male partner have pathogenic variants in the same gene, matching is made by a blinded process. CGT 600 highlights gene matching to select an egg donor [4, 5], but does not detail the process. Autosomal recessive disorders are by far the largest group, but X-linked disorders represent the largest relative burden. X-linked conditions make up about 5% of genes tested but represent 40% of disease risk in IVI’s ART pregnancies [5]. Males have only one gene copy for X-linked recessive traits and show complete penetrance of pathogenic variants. Egg donors are excluded from participation in the egg-donation program if an X-linked pathogenic or likely pathogenic variant is detected [4].

Igenomix preconception genetic screening programs described on IVI’s website reflect published procedures [4, 5]. However, the English CGT 600 consent information is confusing and can be misunderstood. The text contains repetitions and instead of clarifying variant detection confuses it. The consent form describes in one paragraph extensive screening of a large number of genes and lists all genes investigated in an included Appendix. Later it describes how variants are detected in the genes listed, also detailing a small range of more common variants in seven genes. Limitations include a number proper for prospective genetics like de novo mutations in the fetus and gonadal mosaicism.

Reading the consent form will make clients anticipate a thorough screening of genes included, giving a high sensitivity and a high probability of identifying disease-causing *ATP7A* variants, except CNV. However, the genetic report surprisingly stated that egg-donor’s *ATP7A* variant had not been reported before (novel) and therefore was not part of Igenomix screening program, although the variant is located in a gene region covered by the CGT 600 test. The egg donor was demonstrated to be a carrier on a blood sample, excluding the possibility of a de novo mutation or gonadal mosaicism.

When informing IVI about the diagnostic failure, they stated that “the CTG test found no evidence of Menkes disease, because the variant detected had not been described as pathological in scientific databases (ClinVar and HGMD). Therefore, the variant was not identified as disease-causing. The current scientific evidence states that the variant would be classified just as likely pathogenic”. This is contradictory and gives no explanation for the misclassification of a class 4 frameshift variant that normally will show up in routine detection strategies. IVI claimed that variant analysis was confined to the limited number of *ATP7A* variants listed on their website.

The *ATP7A* pathogenic variant profile includes a high proportion of exon deletions and duplications (CNV) [11] that will not be detected, while point mutations ~ 80% (missense, splice site, nonsense, and small frameshift deletions/insertions) should be detected by CGT 600, making scoring the significant step. Frameshift variants should be classified as pathogenic or likely pathogenic (classes 4 and 5), and according to Igenomix, lead to exclusion of the egg donor [4].

Igenomix, Spain lists ten *ATP7A* variants (v1.1), while the Brazilian branch lists 68. Of variants listed on the Spanish website, three are known to cause Menkes disease (c.1639C>T; c.2938C>T; c.3294+2T>G) while two variants lead to milder forms, SMAX3 and OHS, respectively (c.2981C>T; c.3911A>G). Two are not pathogenic (c.2531G>A; c.3931A>G), and three frameshift variants (c.1974_1977dupGTTT; c.3257_3258delAC; c.3915_3921delCTCCCCA) were not reported earlier. The two polymorphisms have later been removed from the list.

We will emphasize that while the entire list of genes is included in the Appendix, a list of specific *ATP7A* variants is not. Extensive search on IVI’s website to find *ATP7A* variants cannot be expected by lay people. It requires professional genetic insight to understand the difference between analysis of a gene or analysis of specific variants in a gene.

How variant examples are selected is unclear, and some are unknown and not recorded in neither ClinVar nor HGMD. The few listed have possibly been found during genetic screening. Notably, none of the unreported variants has been submitted to the databases used to evaluate pathogenicity. In ClinVar, gene variants are disease scored by submitters, and pathogenicity should be used with caution [22]. ClinVar may potentially be used to detect benign variants (polymorphisms) without clinical significance, and this is how Igenomix advise to use the database [4, 5]. HGMD/*ATP7A* is a database listing pathogenic variants from clinically verified Menkes patients. Currently, it contains about 400, and selection of only ten variants is far from current standards. Most *ATP7A* pathogenic variants are private [11] and will unlikely be filed in any database. However, HGMD/*ATP7A* contains similar copy-count changes within the same gene region as M’s variant [11, 23].

It is not sufficient to limit the detection strategy to a small range of variants, and two of these variants listed by Igenomix are even without proven pathogenicity. The technology used by Igenomix allows analysis of the whole gene instead of selected variants within a gene. Surprisingly this failed to detect the *ATP7A* variant observed in the present case. Full CGT 600 screening is also used on the male partner, and if care is not taken, the X-linked *ATP7A* gene may be matched as the autosomal recessive *ATP7B* gene with risk of misdiagnosis. The egg donor has an *ATP7B* variant of uncertain significance, NM_000053.3: c.4301C>T, p.Thr1434Met, a finding that could potentially lead to confusion between *ATP7A* and *ATP7B* in the matching process.

The IVI Igenomix company holds an ISO 15189 certification that requires timely delivery of accurate and reliable results, including qualified genetic skills to evaluate correctly. However, this certification did not ensure the identification of a likely pathogenic variant in the *ATP7A* gene, and the reason will require thorough evaluation of their screening strategy. Genetic web counseling was offered after disclosure of a likely pathogenic *ATP7A* variant in the egg donor, shortly before term of a potentially affected boy. No psychological follow-up was offered to support the couple nor other forms of compensation. This is not in line with professional support provided at governmental hospitals. Furthermore, the medical aspects of Menkes disease such as medication and copper therapy were not supported by the company.

There are no international guidelines or regulations for screening and counseling of neither gamete donors nor recipients, but the field is currently being discussed. Opposed to national hospital-based laboratories, no international regulations exist for private firms. This should be addressed in an open, official, and unbiased discussion, and international legislation may need adjustment. Possibly there should be a legal requirement for liability insurance to cover accidental misdiagnosis.

The egg donor is now blocked internationally to prevent further use in ART. The case emphasizes that genetic screening procedures need thorough and critical evaluation by private companies comparable to governmental hospital laboratories. Furthermore, the counseling and support to families need to be adequate and adhere to international standards for good clinical practice. Genetic counseling related to all genetic findings is paramount. This report demonstrates that a private company has made a mistake concerning a genetic diagnosis and this unfortunate case should be taken very seriously with the aim of preventing such tragedies in the future.

For heritable diseases, accurate genetic risk assessment has fundamentally changed because of massive technological advances [24–26]. These improvements are of major impact for couples who want to minimize the risk of having babies with serious genetic disorders. But using these genetic technologies is not without caveats because all human beings harbor numerous genetic variants where interpretation of pathogenicity is a non-trivial task. In ART, major steps have been taken from retrospective to prospective evaluation, implying new ethical considerations. There is a significant difference between finding the gene that is mutated in a sick child and screening for a possible pathogenic gene variant in a number of genes, where none needs to be affected. Variant evaluation criteria are sharp and sufficiently well defined, and the staff should be well trained [27–29]. This also calls for consent forms that can be understood by lay people so they recognize the limitations of the tests. Companies have an ethical obligation when embarking on preconception screening of gamete donors [30].
